# Alarmone ppGpp modulates bacterial motility, zeamine production, and virulence of *Dickeya oryzae* through the regulation of and cooperation with the putrescine signaling mechanism

**DOI:** 10.1128/msphere.00682-24

**Published:** 2025-03-20

**Authors:** Zurong Shi, Zhibin Liang, Qian Yang, Lian-Hui Zhang, Qingwei Wang

**Affiliations:** 1 School of Biological Engineering, HuaiNan Normal University, Huainan, China; 2 Guangdong Laboratory for Lingnan Modern Agriculture, Guangdong Province Key Laboratory of Microbial Signals and Disease Control, Integrative Microbiology Research Centre, South China Agricultural University, Guangzhou, China; 3 School of Medicine, Anhui University of Science and Technology, Huainan, China; University of Kentucky College of Medicine, Lexington, Kentucky, USA

**Keywords:** putrescine, zeamines, motility, transport, virulence

## Abstract

Putrescine is an important interspecies and interkingdom communication signal, modulating the bacterial motility, biofilm formation, and virulence of *D. oryzae*. The understanding of the regulation of putrescine biosynthesis and transport in *D. oryzae* is limited. In this study, we report that alarmone ppGpp hierarchically modulates putrescine biosynthesis and transport and synergistically cooperates with putrescine to regulate virulence traits and the virulence of *D. oryzae*. We found that the alarmone ppGpp synthesized by RelA regulated putrescine biosynthesis through modulating *speA* expression, and the product putrescine would thus inhibit the expression of *potF* and *plaP*. Remarkably, we unveiled the synergistic effect of alarmone ppGpp and putrescine on the modulation of swimming motility and zeamine production. Compared with the single deletion of either *relA* or *speA*, the double deletion of *relA* and *speA* could decrease the expression of RNA chaperone encoded gene *hfq* and the production of phytotoxin zeamine, which further attenuated the capability of *D. oryzae* EC1 in inhibition of rice seed germination. Collectively, the findings from this study depict alarmone ppGpp regulation on putrescine biosynthesis and transport and present the cooperation of regulation of alarmone ppGpp and putrescine in the virulence of *D. oryzae*.

**IMPORTANCE:**

*Dickeya oryzae* is the causal agent of rice root rot disease. Bacterial motility and phytotoxic zeamines are characterized as two major virulent factors during *D. oryzea* infecting rice seed. Putrescine, as an interspecies and interkingdom communication signal for the infections of *D. oryzae*, has been previously demonstrated to be involved in the modulation of bacterial motility. Here we report the novel synergistic effect of putrescine signal and alarmone ppGpp on the regulation of both zeamine production and bacterial motility via modulating the expression of RNA chaperone-encoded gene *hfq*. In addition, we also showed that alarmone ppGpp hierarchically modulates putrescine biosynthesis and transport. Therefore, the findings of this study unveil the previously undetermined contribution of putrescine in the modulation of virulence determinants, and the regulatory mechanism of putrescine biosynthesis and transport in *D. oryzae*.

## INTRODUCTION

Most members of the genus *Dickeya* are phytopathogenic bacteria causing either soft rots or vascular wilts in their plant hosts (1–4). These devastating plant pathogens can infect numerous economic and food crops and ornamental plants causing significant impacts on agriculture; therefore, genus *Dickeya* was classified among the top 10 most important bacterial plant pathogens (5–8). Several species of this genus, that is, *Dickeya oryzae*, have a broad host range capable of infecting both monocot and dicot plants (1, 9, 10). *D. oryzae*, the causal agent of rice foot rot disease in Asian and European countries, was reclassified as a heterogeneous species from *D. zeae* recently (11). Similar to other species of *Dickeya*, *D. oryzae* can cause significant soft rot symptoms during interaction with its plant hosts due to the action of a larger number of plant cell wall degrading enzymes (PCWDEs) (12–14). In addition, bacterial motility and phytotoxic zeamines are characterized as two major virulent determinants during *D. oryzea* invasion of rice seed (15–18). Our previous works unveiled that bacterial motility and production of phytotoxin zeamines of *D. oryzea* EC1 strain were controlled by AHL and Vfm quorum sensing system, putrescine signaling system, secondary messenger cyclic di-GMP (c-di-GMP), transcriptional regulators, that is, SlyA, Fis, and OhrR, two-component system TzpS-TzpA, and RNA chaperone Hfq (12, 14–17, 19–21). Among them, the putrescine signaling system is involved in the modulation of bacterial motility while RNA chaperone Hfq could regulate both bacterial motility and zeamine production in *D. oryzae* (17, 21).

Putrescine is an aliphatic hydrocarbon molecule with quaternary nitrogen groups present in all living cells (22, 23). The intracellular homeostasis of putrescine is tightly regulated by its synthesis, catabolism, and transport. Two pathways are used for the formation of putrescine, which depends on the initial substrate. One is named ODC, which starts with ornithine, and production of putrescine relies on ornithine decarboxylation catalyzed by SpeC protein, and the other starts with arginine, which is catalyzed by an arginine decarboxylase SpeA to form agmatine, and this is thus converted to putrescine by agmatine ureohydrolase SpeB protein (24). Despite the fact that the two anabolic pathways are highly conserved in bacteria, there is evidence that they make different contributions to putrescine homeostasis in different bacterial species. For instance, the ODC pathway is essential for putrescine biosynthesis, while the other pathway appears to play complementary roles in maintaining intracellular putrescine levels of *Escherichia coli* (24). However, in *D. oryzae*, we found that the SpeA-dependent pathway is crucial to putrescine biosynthesis and signaling in modulating bacterial motility and virulence, whereas the ODC pathway mediated by SpeC is not required (17). Furthermore, several factors have been found affecting the activities of the two key enzymes in anabolic pathways of putrescine in bacteria. The protein activity of SpeC is greatly activated or inhibited by a variety of nucleotides, including GTP, cyclic AMP (cAMP), and (p)ppGpp (25, 26), whereas the activity of SpeA is repressed by cAMP (27), and expression of SpeA can be repressed by putrescine and spermidine (28). In addition, transport systems of PotFGHI and PlaP are involved in putrescine incorporation in several gram-negative bacteria including *D. oryzae* (17, 29). However, the regulons of SpeA-dependent putrescine biosynthesis and its transport in *D. oryzae* are still unclear.

(p)ppGpp, known as a stress alarmone, is produced by proteins belonging to the RelA/SpoT homolog family (RSH) of bacteria under nutrient stress, that is, amino acid or fatty acid starvation (30–32). RelA is the (p)ppGpp synthetase I or GTP-pyrophosphokinase that synthesizes (p)ppGpp from GTP/GDP and ATP, whereas SpoT is a bifunctional (p)ppGpp synthetase II or pyrophosphohydrolase (33, 34). However, (p)ppGpp levels in bacteria are not only involved in responses to nutrient stress but also can accumulate during exposure to a wide range of signals, including oxygen variation, pH downshift, and darkness (35–38). Furthermore, alarmone (p)ppGpp can regulate the pathogenicity of plant pathogenic bacteria by modulation of quorum sensing signal degradation, cell wall-degrading enzyme production, and type III secretion system expression (39–42).

In this study, we report that the putrescine synthesized by SpeA could inhibit the expression of two major putrescine transport system gene *potF* and *plaP*, and alarmone ppGpp synthesized by RelA could regulate *potF* and *plaP* expression via modulation of *speA* expression. Interestingly, it was demonstrated that alarmone ppGpp and putrescine cooperatively regulate bacterial motility and zeamine production by modulating the expression of RNA chaperone-encoded gene *hfq*. Putrescine serves as an interspecies and interkingdom communication signal for the infections of *D. oryzae*. Understanding the regulatory mechanism of putrescine biosynthesis and transport and the previously undetermined contribution of putrescine in the modulation of virulence determinants would be of significance to control the rice foot rot disease caused by *D. oryzae*.

## RESULTS

### Putrescine synthesis by *speA* inhibited the expression of putrescine uptake systems PotFGHI and PlaP in *D*. *oryzae* EC1

**Fig 1:**
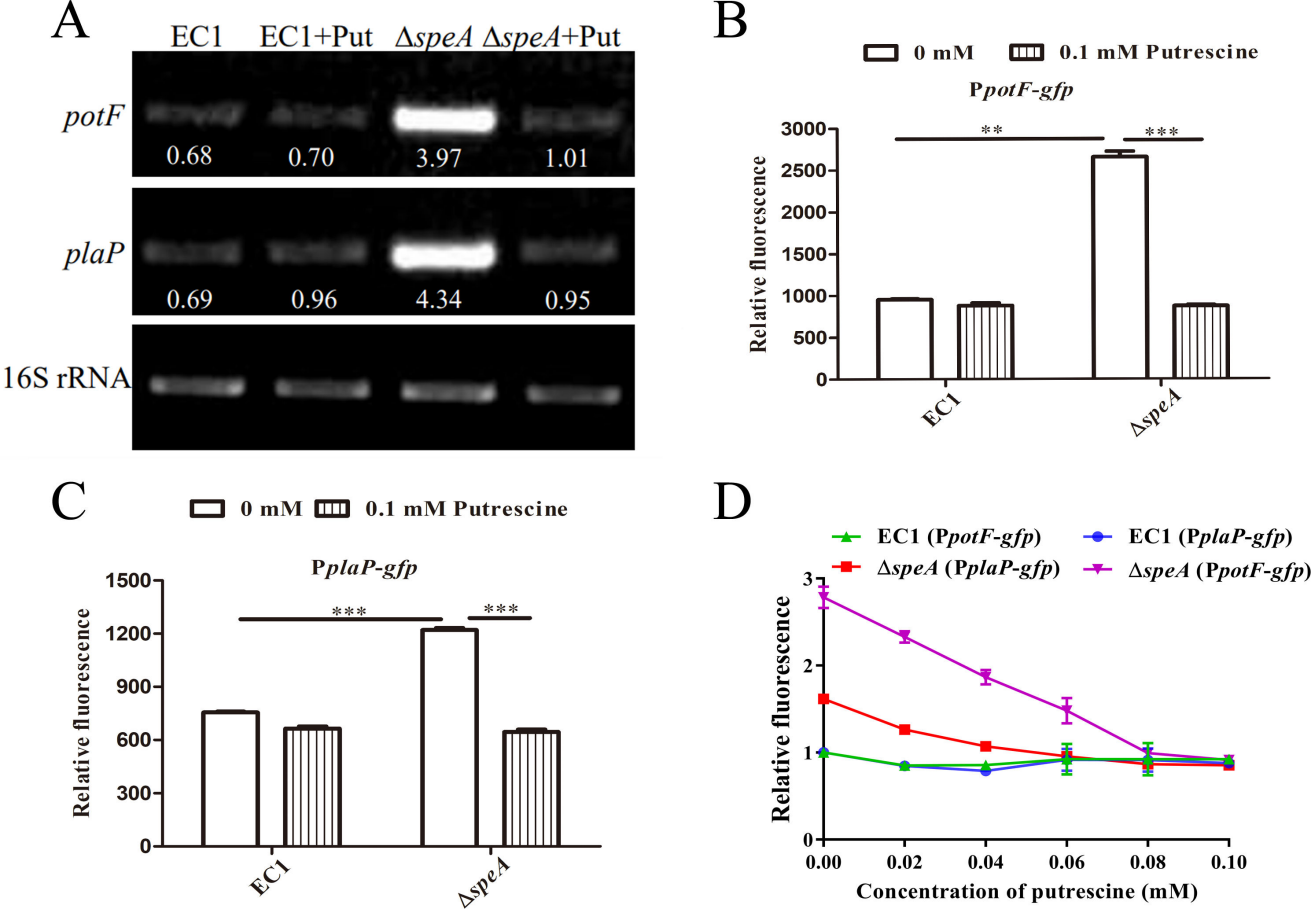
Mutation of the *speA* gene of *Dickeya oryzae* resulted in increased expression of genes *potF* and *plaP*. (**A**) RT-PCR analysis of putrescine signal on modulating *potF* and *plaP* expression in the strains EC1 and ∆*speA* with or without exogenous addition of putrescine, respectively; and at the same cultured conditions, the effects of putrescine signal on *potF* (**B**) and *plaP* (**C**) gene expression were also measured assessing relative fluorescence of the *potF-gfp* and *plaP-gfp* transcriptional fusions. (**D**) The susceptibility of genes *potF* and *plaP* to different concentrations of putrescine signal. The reference gene of 16S rRNA was used to standardize the samples of RNA. EC1 and ∆*speA* indicated the wild-type strain and *speA* gene deletion mutant of *Dickeya oryzae*, respectively; EC1 + Put and ∆*speA* + Put indicated the strains of EC1 and ∆*speA* cultured in swimming motility medium with putrescine signal at final concentration of 0.1 mM, respectively. The data shown are the means ± SE (*n* = 3). ** and ***, corrected *P* values of <0.05 and <0.01, respectively.

Our previous work unveiled that PotFGHI and PlaP contributed to the uptake of extracellular putrescine and the putrescine signaling in bacterial motility and biofilm formation in *D. oryzae* EC1 while bacterial putrescine biosynthesis gene *speA* was abolished (17). To investigate whether the expression of putrescine uptake systems could be affected by putrescine molecule, the expression level of *potF* and *plaP* in wild-type strain EC1 and a *speA* in-frame deletion mutant, that is, ∆*speA*, were first measured by semiquantitative reverse transcription-PCR (RT-PCR). The results indicated that the transcript levels of *potF* and *plaP* in ∆*speA* were 5.8 and 6.3-fold higher than those in wild-type strain EC1, respectively (Fig. 1A), and exogenous addition of putrescine at a final concentration of 0.1 mM could decrease the transcript levels of *potF* and *plaP* in ∆*speA* comparable to those in wild-type strain EC1 (Fig. 1A). The suppressive effect of putrescine on *potF* and *plaP* expression was also validated in the reporter strain assay. The *gfp* reporter constructs, that is, P*potF-gfp* and P*plaP-gfp,* in which the *gfp* was placed under the control of the *potF* and *plaP* promoters, respectively, were transformed into wild-type strain EC1 and mutant ∆*speA*, and the relative fluorescence EC1(P*potF-gfp*), ∆*speA*(P*potF-gfp*), EC1(P*plaP-gfp*), and ∆*speA*(P*plaP-gfp*) were measured and compared under the culturing conditions with or without exogenous additional putrescine. The results indicated that exogenous addition of putrescine at a final concentration of 0.1 mM decreased the relative fluorescence intensity of ∆*speA*(P*potF-gfp*) and ∆*speA*(P*plaP-gfp*) to a level comparable to those of EC1(P*potF-gfp*) and EC1(P*plaP-gfp*), respectively, under the culturing conditions supplemented with or without putrescine (Fig. 1B and C). In addition, we found that *potF* and *plaP* expression in mutant ∆*speA* were inhibited by the exogenous addition of putrescine in a dosage-dependent manner from 0.02 to 0.1 mM. Exogenous addition of putrescine at a final concentration of 0.06 and 0.08 mM could decrease the relative fluorescence intensity of ∆*speA*(P*plaP-gfp*) and ∆*speA*(P*potF-gfp*) to a level comparable to those in EC1(P*plaP-gfp*) and EC1 (P*potF-gfp*) supplied with the same amount of putrescine, respectively (Fig. 1D). All these findings indicated putrescine signal synthesis by *speA* negatively regulates the transcriptional expression of putrescine uptake systems PotFGHI and PlaP in *D. oryzae* EC1.

### Alarmone ppGpp synthesized by RelA repressed the transcriptional expression of *potF* and *plaP*

To characterize the regulatory mechanism of putrescine on *potF* and *plaP* expression, the reporter strain EC1 (P*potF-gfp*) was mutated with mariner-based transposon carried by pBT20, and the relative fluorescence of transposon insertion mutants was measured to identify the genes affecting *potF* expression. A total of 15,000 mutants were screened, and a transposon insertion mutant T1536, which harbored a transposon insertion at the 1,205th base pair of a 2,235 bp coding sequence of the (p)ppGpp biosynthesis gene *relA* (NCBI accession no. AJC67484.1), represented a higher level of relative fluorescence intensity than EC1 (P*potF-gfp*).

**Fig 2:**
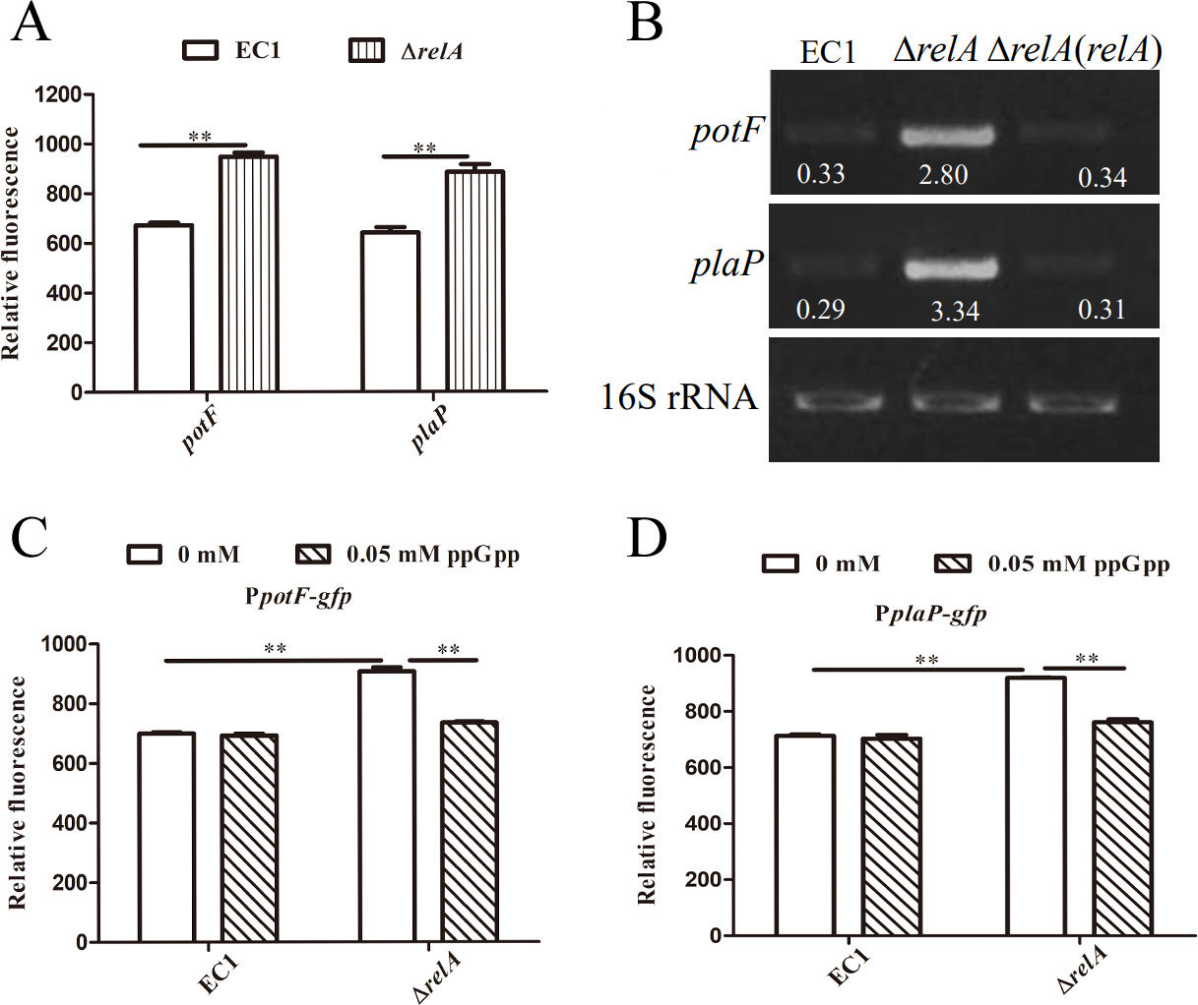
RelA depletion caused increased expression of genes *potF* and *plaP* in *Dickeya oryzae*. (**A**) The effects of RelA on genes *potF* and *plaP* expression were measured by assessing the relative fluorescence of the *potF-gfp* and *plaP-gfp* transcriptional fusions in the wild-type EC1 and ∆*relA* strains. (**B**) RT-PCR analysis of RelA on regulation genes *potF* and *plaP* expression. The effects of alarmone ppGpp on *potF* (**C**) and *plaP* (**D**) gene expression were measured by assessing the relative fluorescence of the *potF-gfp* and *plaP-gfp* transcriptional fusions. The reference gene of 16S rRNA was used to standardize the samples of RNA. EC1, ∆*relA,* and ∆*relA*(*relA*) indicated the wild-type strain, *relA* deletion mutant, and the complemented strain of mutant ∆*relA*, respectively. The data shown are the means ± SE (*n* = 3). **, corrected *P* value of <0.05.

To further assess the involvement of *relA* in the regulation of *potF* and *plaP* expression, the reporter strains ∆*relA*(P*potF-gfp*) and ∆*relA*(P*plaP-gfp*) were constructed by introducing the *gfp* reporter constructs P*potF-gfp* and P*plaP-gfp* into the *relA* in-frame deletion mutant and their relative fluorescence was measured and compared to those of the wild-type strain EC1 with the same reporter constructs, respectively. The results indicated that ∆*relA*(P*potF-gfp*) and ∆*relA*(P*plaP-gfp*) had a higher amount of relative fluorescence than EC1(P*potF-gfp*) and EC1(P*plaP-gfp*), respectively (Fig. 2A). Similarly, the RT-PCR analysis also indicated the transcriptional levels of *potF* and *plaP* in ∆*relA* were significantly higher than those in wild-type strain EC1 and the complemented strain ∆*relA*(*relA*) (Fig. 2B). These findings suggest that *relA* is implicated in the regulation of *potF* and *plaP* expression. RelA was characterized as an alarmone (p)ppGpp synthetase in bacteria (33, 34). To further determine the role of ppGpp on the regulation of *potF* and *plaP* expression, the expression levels of *potF* and *plaP* were determined by reporter strains, ∆*relA*(P*potF-gfp*) and ∆*relA*(P*plaP-gfp*), after exogenous addition of ppGpp. The results indicated that the expression level of *potF* and *plaP* in ∆*relA* significantly reduced after an exogenous supplement of 0.05 mM ppGpp, which represented a comparable level with those in wild-type strain EC1, respectively (Fig. 2C and D). All these findings indicated that the alarmone ppGpp synthesized by RelA repressed the expression of putrescine uptake systems genes *potF* and *plaP* in *D. oryzae* EC1.

### Alarmone ppGpp regulated *potF* and *plaP* expression through modulation of *speA* expression

**Fig 3:**
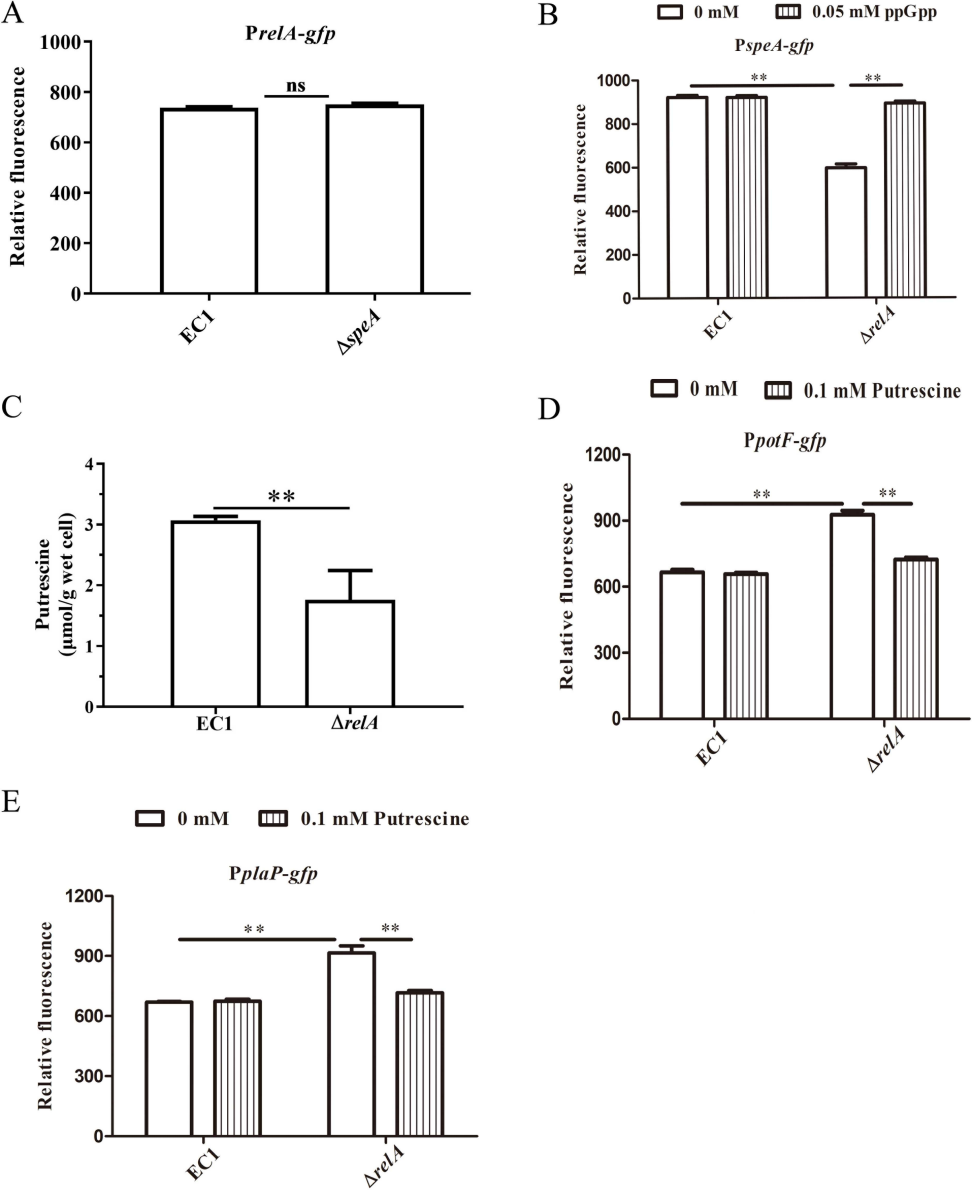
RelA depletion caused decreased expression of gene *speA* of *Dickeya oryzae*. (**A**) The relative fluorescence of the *relA-gfp* transcriptional fusion in the wild-type EC1 and ∆*speA* strains. (**B**) The analysis of alarmone ppGpp on the regulation gene *speA* expression and intracellular concentration of putrescine (**C**). (**D**) The effects of putrescine signal on *potF* and *plaP* expression were measured by assessing the relative fluorescence of the *potF-gfp* and *plaP-gfp* transcriptional fusions in the wild-type EC1 and ∆*relA* strains (**E**), respectively. **, corrected *P* value of <0.05.

Given that expression of putrescine uptake system genes *potF* and *plaP* could be repressed by the alarmone ppGpp synthesized by *relA* and putrescine synthesized by *speA* in *D. oryzae* EC1, we proposed that a regulatory linkage between biosynthesis of ppGpp and putrescine contributes to the regulation of *potF* and *plaP* expression in *D. oryzae* EC1. To validate this hypothesis, the relative fluorescence of the *gfp* reporter strains, EC1(P*relA-gfp*) and ∆*speA*(P*relA-gfp*), was compared to conduct the effect of putrescine biosynthesis on *relA* expression, and the effect of ppGpp biosynthesis on *speA* expression was also conducted following the similar method using the *gfp* reporter strains, EC1(P*speA-gfp*) and ∆*relA*(P*speA-gfp*). The results indicated that the relative fluorescence of EC1(P*relA-gfp*) and ∆*speA*(P*relA-gfp*) was comparable (Fig. 3A), whereas the relative fluorescence was significantly reduced in EC1(P*speA-gfp*) compared to ∆*relA*(P*speA-gfp*) (Fig. 3B), which could be recovered by exogenous supplement of ppGpp at a final concentration of 0.05 mM. Importantly, the intracellular concentration of putrescine in mutant ∆*relA* was about twofold lower than that in the wild-type strain (Fig. 3C). These findings suggested that the alarmone ppGpp synthesized by *relA* regulates *speA* expression to modulate intracellular putrescine contents in *D. oryzae* EC1. Consistently, we found putrescine could affect the regulation of alarmone ppGpp on *potF* and *plaP* expression. An exogenous supplement of putrescine at a final concentration of 0.1 mM reduced the relative fluorescence intensity of ∆*relA*(P*potF-gfp*) and ∆*relA*(P*plaP-gfp*) to their wild-type forms, respectively (Fig. 3D and E). The above findings indicated that the ppGpp synthesized by RelA modulates *potF* and *plaP* expression through the regulation of *speA* expression for putrescine biosynthesis.

### Alarmone ppGpp regulated bacterial cell motility through modulation of putrescine signaling

**Fig 4:**
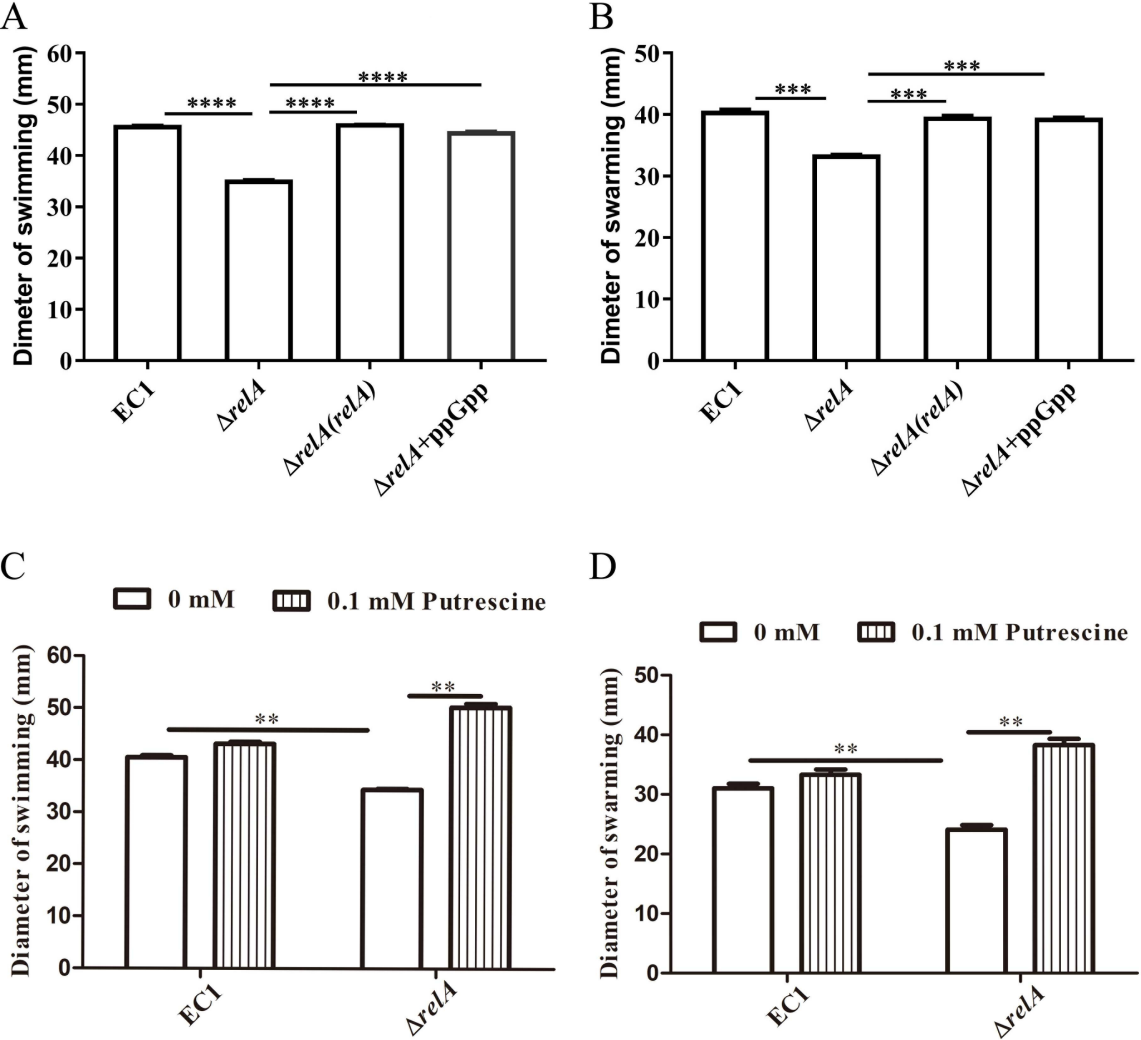
Disruption of *relA* decreased bacterial cell motility of *Dickeya oryzae*. (**A**) The swimming and swarming (**B**) motility of the *relA* deletion mutant significantly decreased compared with the wild-type EC1 and its complemented strain, and exogenous addition 0.05 mM alarmone ppGpp could rescue these cell motility to the levels of wild-type stain, respectively. (**C**) The decreased swimming and swarming (**D**) motility of the *relA* deletion mutant was rescued by exogenous addition 0.1 mM putrescine signal, respectively. The data shown are the means ± SE (*n* = 3). ****, corrected *P* value of <0.0001. ***, corrected *P* value of <0.001. **, corrected *P* value of <0.05.

Our previous work unveiled the significant contribution of putrescine on bacterial swimming and swarming motility (17). We hypothesized alarmone ppGpp regulation on *speA* expression also contributes to swimming and swarming motility in *D. oryzae* EC1. To assess the role of ppGpp in bacterial swimming and swarming motility, the swimming and swarming motility of wild-type strain EC1 and mutant ∆*relA* were compared on semisolid agar plates. The results indicated that the inactivation of *relA* compromised the bacterial swimming and swarming (Fig. 4A and B), and these could be restored by *in trans* expression of *relA* (Fig. 4A and B). In addition, the defective swimming and swarming motility of mutant ∆*relA* could be rescued by the exogenous addition of not only ppGpp but also putrescine at a final concentration of 0.1 mM, respectively (Fig. 4C and D). Collectively, these findings suggested that alarmone ppGpp regulates the swimming and swarming motility by modulating the putrescine signaling pathway in *D. oryzae* EC1.

### Alarmone ppGpp and putrescine had a synergistic effect on modulation of bacterial cell motility and zeamine production

**Fig 5:**
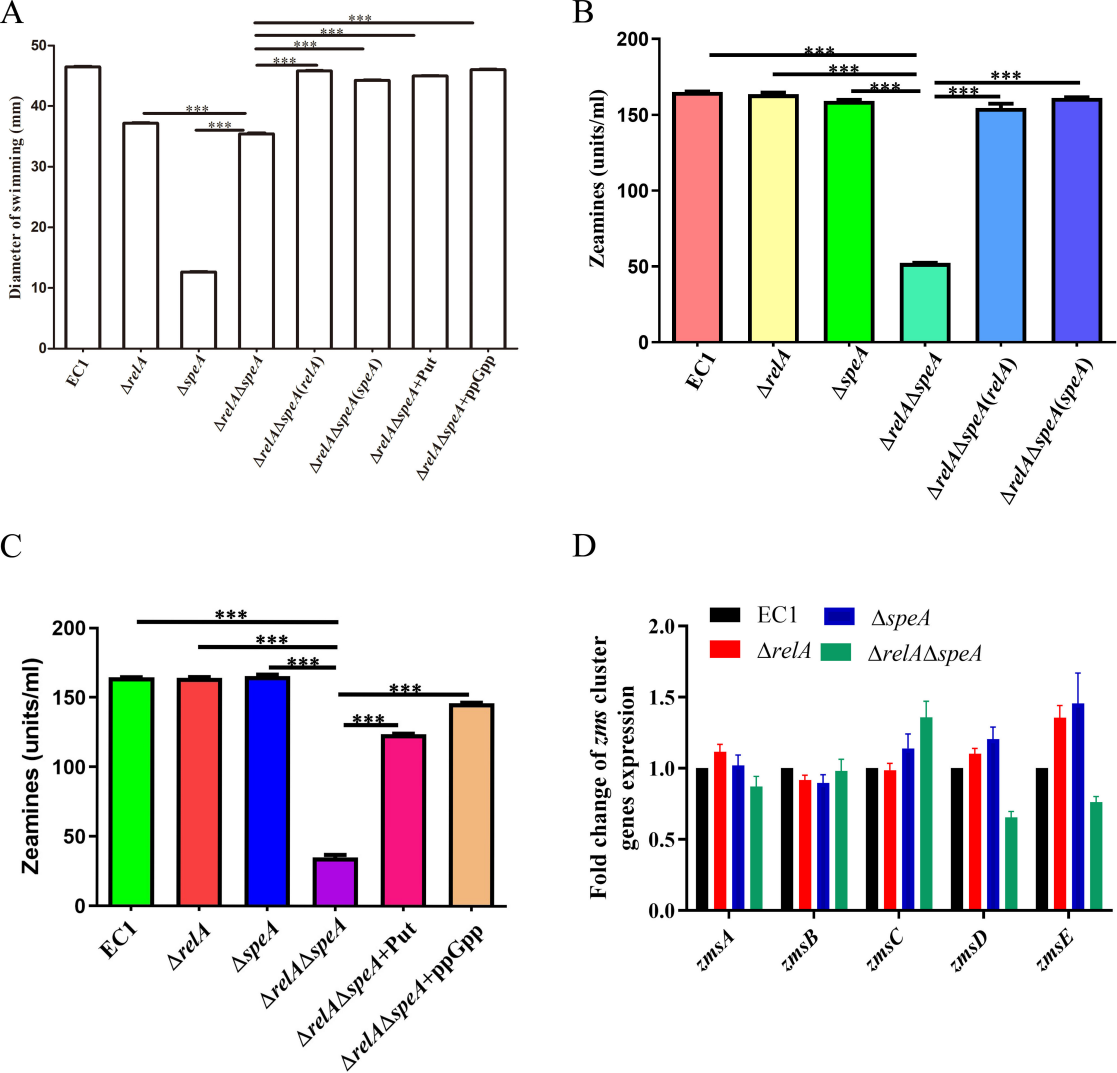
Putrescine signal and alarmone ppGpp synergistically regulated zeamine production and swimming motility of *Dickeya oryzae*. (**A**) Effects of genes *relA* and *speA* double disruption on the bacterial swimming motility. (**B**) Statistical analysis of zeamine production of wild-type EC1, the double deletion mutant of genes *relA*, *speA,* and its complementary strains. (**C**) The decreased zeamine production of genes *relA* and *speA* double deletion mutant was rescued by exogenous addition putrescine signal or alarmone ppGpp, respectively. (**D**) Effects of genes *relA* and *speA* double disruption on the *zms* genes transcript levels. ∆*relA*∆*speA* indicated *relA-speA* double disrupted mutant, ∆*relA*∆*speA*(*relA*) and ∆*relA*∆*speA*(*speA*) indicated the complemented strains of mutant ∆*relA*∆*speA*, and ∆*relA*∆*speA +* Put and ∆*relA*∆*speA +* ppGpp indicated mutant ∆*relA*∆*speA* cultured with exogenous addition of 100 nM putrescine signal and 50 nM alarmone ppGpp, respectively. The data shown are the means ± SE (*n* = 3). ***, corrected *P* value of <0.001.

The above findings showed that the alarmone ppGpp modulates putrescine biosynthesis, conferring the regulation of *potF* and *plaP* expression and bacterial cell motility in *D. oryzae* EC1. In addition to the hierarchical regulatory relationship from ppGpp to putrescine biosynthesis, we further investigate whether there is the synergistic effect of ppGpp and putrescine on the regulation of virulence traits. We determined the impact of the simultaneous knockdown of *relA* and *speA* on bacterial growth and bacterial swimming motility and found that simultaneous inactivation of *relA* and *speA* would not affect the growth of *D. oryzae* EC1 (Fig. S1) but decrease bacterial swimming (Fig. 5A). Expression of either *relA* or *speA* and exogenous addition of either putrescine or ppGpp in *relA-speA* double deletion mutant restore bacterial swimming motility (Fig. 5A). These findings suggested the synergistic effect of ppGpp and putrescine on the regulation of swimming motility. In addition, we also noticed that the swimming motility apparently increased in *relA-speA* double deletion mutant compared to ∆*speA*, which suggested that *relA-speA* double deletion affected other signaling pathways modulating swimming motility in *D. orzyae* EC1.

In addition, the synergistic effect of ppGpp and putrescine regulation was also detected in the production of zeamine phytotoxins, which are key virulence factors of *D. orzyae* EC1 for inhibiting rice seed germination (18, 43). We determined the zeamine production for wild-type strain EC1, mutant ∆*relA* and ∆*speA*, and *relA-speA* double deletion mutant at the time when bacterial cells can produce a great number of zeamines in LS_5_ medium, which was optimized for zeamine production (44). The results showed that the single deletion of either *relA* or *speA* did not affect the zeamine production (Fig. 5B; Fig. S2). However, the simultaneous knockdown of *relA* and *speA* could significantly decrease zeamine production compared to the wild-type strain EC1, which could be rescued by *in trans* expression of either *relA* or *speA* and exogenous addition of either putrescine or ppGpp (Fig. 5B and C; Fig. S2B and C). Consistently, we found that the transcript levels of *zmsD* and *zmsE*, two major zeamine biosynthetic genes in *D. oryzae* EC1 (45), significantly reduced in *relA-speA* double deletion mutant rather than in the *relA* or *speA* single deletion mutant (Fig. 5D). These results showed that ppGpp and putrescine have a synergistic effect on regulation of zeamine production through modulation of *zms* genes expression.

### The synergistic effect of alarmone ppGpp and putrescine was dependent on the regulation of *hfq* on zeamine production

**Fig 6:**
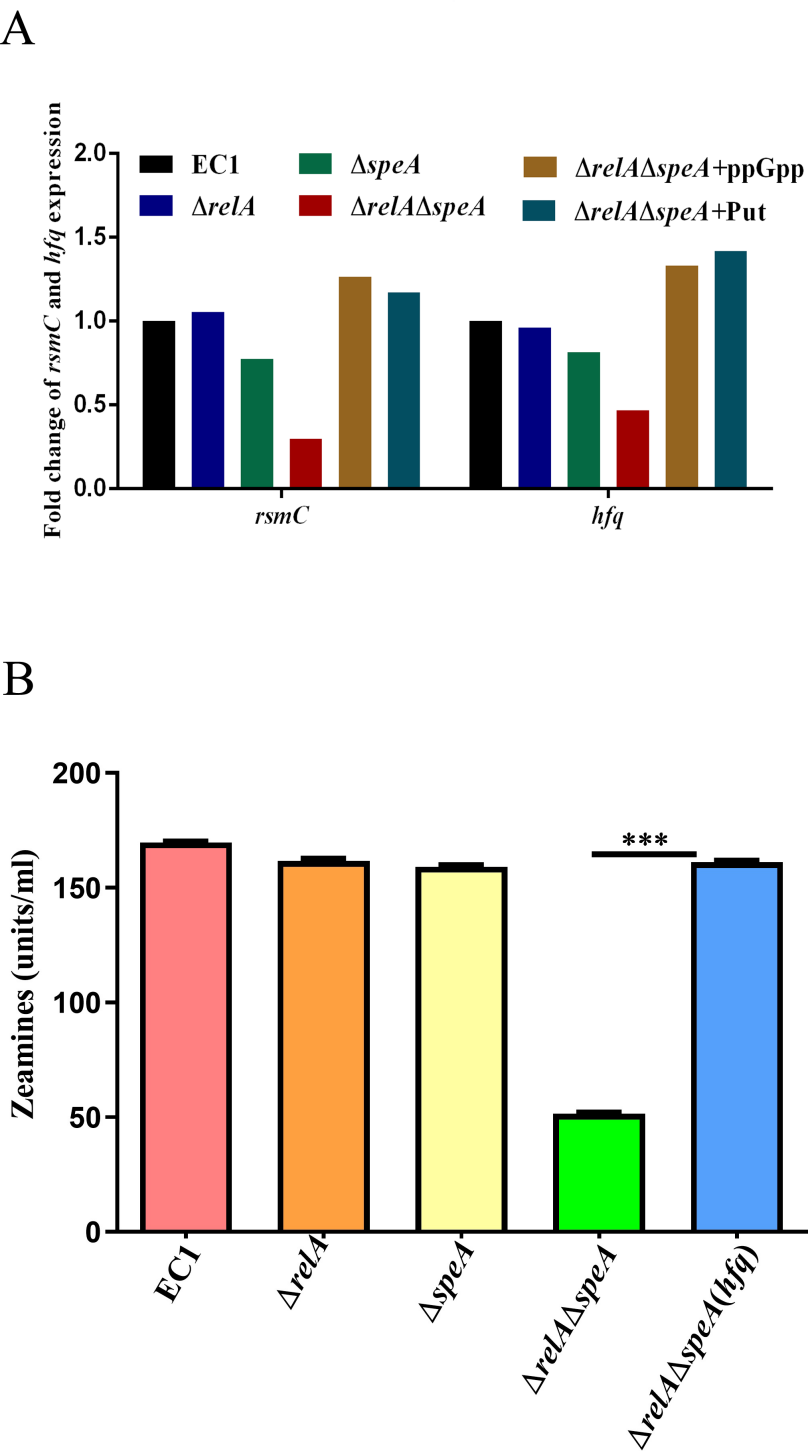
Putrescine signal and alarmone ppGpp in parallel regulated gene *hfq* expression to control zeamine production in *Dickeya oryzae*. (**A**) The transcript levels of genes *rsmC* and *hfq* were significantly reduced in genes *relA* and *speA* double disruption mutant, which were rescued via exogenous addition putrescine signal and alarmone ppGpp, respectively, grown in LS_5_ medium to OD_600_ of 1.5. (**B**) The decreased zeamine production of genes *relA* and *speA* double deletion mutant was rescued by the over-expression of *hfq* gene. ∆*relA*∆*speA* indicated *relA-speA* double disrupted mutant, ∆*relA*∆*speA*(*hfq*) indicated over-expression gene *hfq* strain of mutant ∆*relA*∆*speA*, and ∆*relA*∆*speA +* Put and ∆*relA*∆*speA +* ppGpp indicated mutant ∆*relA*∆*speA* cultured with exogenous addition of 100 nM putrescine signal and 50 nM alarmone ppGpp, respectively. The data shown are the means ± SE (*n* = 3). ***, corrected *P* value of <0.001.

To investigate the ppGpp- and putrescine-dependent regulatory network of zeamine production, we determined the transcript expression of the previously determined regulators of zeamine production. Our recent work underlined that the sRNA chaperone Hfq was implicated in swimming motility and zeamine production in *D. oryzae* EC1 (21). In this study, the reverse transcription-quantitative PCR (RT-qPCR) analysis showed that the expression level of *hfq* in the double deletion mutant ∆*relA*∆*speA* was 1.9-fold less than that in the wild-type strain EC1 (Fig. 6A), which also could be restored by exogenous addition of putrescine and ppGpp (Fig. 6A). In addition, we found that the zeamine production defect of the *relA-speA* double mutant could be rescued by *in trans* expression of *hfq* (Fig. 6B; Fig. S2D). Collectively, these findings unveiled that putrescine and ppGpp signals synergistically regulate the zeamines production via modulating the transcriptional expression of *hfq*.

### Alarmone ppGpp and putrescine controlled the pathogenicity of *D*. *oryzae* EC1 in rice seeds

**Fig 7:**
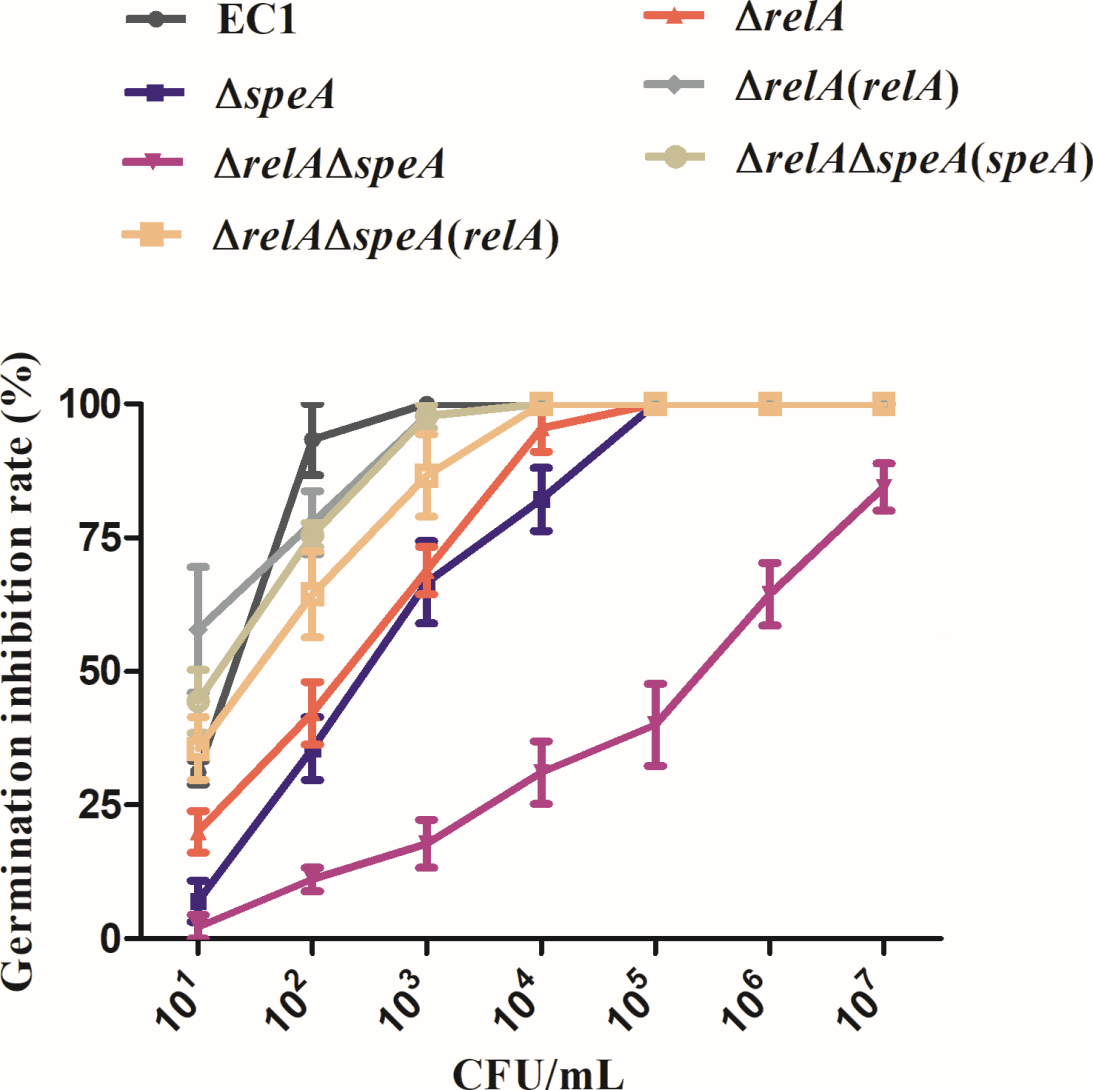
Double deletion of genes *relA* and *speA* attenuated bacterial virulence of *Dickeya oryzae* on rice seeds. Rice seeds were soaked in a bacterial suspension containing various amounts of bacterial cells as indicated for 5 h before being transferred to plates with moisturized filter papers. EC1, ∆*relA*, ∆*speA,* and ∆*relA*∆*speA* indicated the wild-type strain, *relA* deletion mutant, *speA* deletion mutant, and *relA-speA* double disrupted mutant, respectively. ∆*relA*(*relA*), ∆*relA*∆*speA*(*speA*), and ∆*relA*∆*speA*(*relA*) indicated the complemented strain of mutants ∆*relA* and ∆*relA*∆*speA*, respectively. The germination rates were counted 5 days later.

Our previous works indicated that swimming motility and zeamine production are two dominant virulence factors for the virulence of *D. oryzae* EC1 in the inhibition of rice seed germination (15–18). Given that alarmone ppGpp and putrescine played significant roles in bacterial motility (Fig. 4) and had a synergistic effect on bacterial motility and zeamine production (Fig. 5; Fig. S2B and C). We hypothesized that alarmone ppGpp and putrescine modulate the pathogenicity of *D. oryzae* EC1 in rice seeds. Rice seed germination assays were conducted in wild-type strain EC1, mutant ∆*relA*, ∆*speA,* and ∆*relA*∆*speA*. The results indicated that the single deletion of either *relA* or *speA* could decrease bacterial capacity in the inhibition of rice seed germination (Fig. 7), suggesting the roles of alarmone ppGpp and putrescine in the pathogenicity of *D. oryzae* EC1 in rice seeds. In addition, we found that double deletion of *relA* and *speA* could result in a significantly higher degree decrement in rice seed inhibitory rate at each bacterial inoculation concentration compared to the single deletion of either *relA* or *speA*, which unveiled the contribution of the synergistic effect of ppGpp and putrescine in the pathogenicity of *D. oryzae* EC1 (Fig. 7)

## DISCUSSION

Biosynthesis and transport of the chemical signal putrescine affect bacterial motility and the virulence of *D. oryzae* (17). In this study, we documented that alarmone ppGpp regulates the expression of putrescine uptake systems PotFGHI and PlaP by modulating the SpeA-dependent putrescine biosynthesis. In addition, we also showed that the simultaneous inactivation of the biosynthesis genes of alarmone ppGpp and putrescine could reduce bacterial motility, phytotoxin zeamine production, and bacterial virulence on rice seeds through down-regulating *hfq* expression, which depicts the synergistic effect of alarmone ppGpp and putrescine on the regulation of virulence traits and virulence of *D. oryzae*.

The intracellular polyamine homeostasis is important for bacterial survival and virulence (46–49). Our previous study showed that exogenously added putrescine could restore the swimming motility of putrescine biosynthesis gene mutant ∆*speA* through the putrescine uptake systems encoded by *potF* and *plaP* (17). In this study, we found the exogenously added putrescine represses the transcriptional expression of putrescine uptake genes *potF* and *plaP* in mutant ∆*speA*, whereas an exogenous addition of putrescine did not significantly affect *potF* and *plaP* expression on wild-type strain EC1 (Fig. 1). Our findings suggest that *D. oryzae* maintains the intracellular putrescine homeostasis by increasing the expression level of putrescine uptake systems while the intracellular putrescine is a deficiency, and inhibiting the expression of putrescine uptake systems while the intracellular putrescine is rich. Such feedback inhibition mechanism mediated by the polyamine for cellular polyamine homeostasis through the regulation of polyamine uptake systems is also reported in *E. coli*. Spermidine could increase the transcriptional inhibition mediated by spermidine-binding protein PotD on the expression of the spermidine uptake system encoded by *potABCD* (50). In addition, the accumulation of putrescine in *E. coli* cells could inhibit the putrescine uptake efficiency (51). However, in *Proteus mirabilis*, regulation of intracellular polyamine homeostasis did not rely on the feedback inhibition mediated by polyamine as putrescine did not affect the expression of *plaP*, which encoded the major putrescine uptake system in *P. mirabilis* (29). All these suggest bacterial species can adopt different mechanisms for ensuring cellular polyamine homeostasis, and the feedback inhibition mechanism mediated by the polyamine through regulation of polyamine uptake systems is a common strategy adopted by some bacterial species.

The biosynthesis of putrescine in bacteria is commonly dependent on two independent pathways mediated by the ODC SpeC and ADC SpeA. In *E. coli*, the ODC SpeC-dependent pathway plays a major role in putrescine production (24). The results from *in vitro* studies showed that the catalyzed activity of partially purified SpeC protein was inhibited in the presence of alarmone ppGpp (25, 26). However, the results from *in vivo* studies showed that the synthesis and activity of SpeA protein were increased in spite of ppGpp production (26), which may lead to a high amount of putrescine production at the time when the synthesis and activity of SpeC protein were decreased (26, 52). These findings suggested that alarmone ppGpp has a negative effect on the SpeC-dependent pathway but not the SpeA-dependent pathway in *E. coli*. In *D. oryzae*, our previous study indicated that SpeA-dependent pathway plays a major role in putrescine biosynthesis and modulation of bacterial motility (17). In this study, we found that alarmone ppGpp could positively regulate the transcriptional expression of *speA* in a rich medium (Fig. 3B and C). This unveiled a distinct regulatory role of alarmone ppGpp on bacterial putrescine biosynthesis. Regulation of ppGpp on bacterial motility was also reported in another *Dickeya* strain, that is, *D. dadantii* 3937. Inactivation of *relA* compromised the swimming motility of *D. dadantii* 3937 (53). Given the relatively close phylogenetic relationship between *D. dadantii* and *D. oryzae*, alarmone ppGpp may also regulate putrescine production for modulation of swimming motility in *D. dadantii* 3937.

Phytotoxin zeamines are major virulence determinants of *D. oryzae* EC1 for inhibition of rice seed germination (18, 43). Our previous works unveiled that the production of phytotoxin zeamine of *D. oryzae* EC1 was controlled by the Vfm quorum sensing system, transcriptional regulators, that is, SlyA, Fis, and OhrR, two-component system TzpS-TzpA, and RNA chaperone Hfq (12, 14, 16, 19–21). The results from this study hint at the cooperation of alarmone ppGpp and putrescine in the modulation of zeamine production in *D. oryzae* EC1. Deletion of either the ppGpp or putrescine biosynthesis gene, that is, *speA* or *relA*, would not affect zeamine production (Fig. 5B; Fig. S2), whereas double deletion of *speA* and *relA* resulted in a significant decrease in zeamine production (Fig. 5; Fig. S2) and further compromised bacterial virulence in rice seeds (Fig. 7). The defect of zeamine production in *speA-relA* double mutant could be restored by *in trans* expression of either *relA* or *speA* (Fig. 5B; Fig. S2B), or supplemented with the ppGpp and putrescine molecules, respectively (Fig. 5C; Fig. S2C). In addition, we found that the regulation of zeamine production by ppGpp and putrescine relies on their cooperative regulation of the transcriptional expression of RNA chaperone Hfq. The *relA-speA* double deletion mutant bore a lower level of *hfq* expression compared to the single deletion mutant, that is, ∆*speA* and ∆*relA*, and wild-type strain EC1 (Fig. 6A). Consistent with the involvement of Hfq in modulation of zeamine production and bacterial virulence in rice seeds (21), double deletion of *relA* and *speA* decreased the expression of two key *zms* genes, that is, *zmsD* and *zmsE*, and attenuate bacterial ability in inhibition of rice seed germination (Fig. 5D and 7). Expression of *hfq* in *trans* restored the zeamine production for the *relA-speA* double mutant (Fig. 6B; Fig. S3D). The transcriptional regulation of alarmone ppGpp and putrescine on *hfq* expression gives a new insight into the relationship between ppGpp synthetase RelA and RNA chaperone Hfq, with a previous notion that the C-terminal domain of RelA, which is required for protein oligomerization but not for ppGpp biosynthesis, could interact with the Hfq to stabilize the initially unstable complex of RNA bound-Hfq monomer and regulate the expression of RyhB-target genes (54). Notably, although double deletion of *relA* and *speA* decreased bacterial swimming, the *relA-speA* double mutant bore a relatively higher swimming motility compared to the mutant ∆*speA* (Fig. 5A). A plausible explanation is that double deletion of *relA* and *speA* affected other metabolic pathways conferring swimming motility in *D. oryzae* EC1, which requires a further elucidation.

In summary, this study unveils alarmone ppGpp modulates the transcriptional regulation of putrescine uptake systems by controlling the SpeA-mediated putrescine biosynthesis in *D. oryzae* EC1 and underlines the importance of alarmone ppGpp and putrescine cooperation on the regulation of Hfq-dependent bacterial virulence. Given that alarmone ppGpp and putrescine are widely conserved chemical signals among bacterial species, it is intriguing to elucidate the significance of their regulatory relationship and cooperation in the modulation of bacterial physiology and metabolism in other bacterial species.

## MATERIALS AND METHODS

### Bacterial strains, plasmids, and reagents

The bacterial strains and plasmids used in this study are listed in Table S1. *D. oryzea* EC1 and its derivatives were grown at 28°C in LB medium unless otherwise stated. Minimal medium [K_2_HPO_4_ 10.5 g/L, KH_2_PO_4_ 4.5 g/L, (NH)_2_SO_4_ 2 g/L, MgSO_4_.7H_2_O 0.2 g/L, FeSO_4_ 0.005 g/L, CaCl_2_ 0.01 g/L, MnCL_2_ 0.002 g/L, glycerol 2 g/L, and mannitol 2 g/L] was used for comparison of bacterial growth rate. LS_5_ medium (K_2_HPO_4_ 5.25 g/L, KH_2_PO_4_ 2.25 g/L, sucrose 10.0 g/L, NH_4_NO_3_ 3.6 g/L, KCl 1.0 g/L, and MgSO_4_·7H_2_O 0.25 g/L, pH 7.0) was used for zeamine production. Putrescine and ppGpp were purchased from Sigma-Aldrich. Antibiotics were added at the following final concentrations when required: kanamycin (Km), 100 µg/mL; streptomycin (Str), 50 µg/mL; and ampicillin (Amp), 50 µg/mL.

### Mutants construction and complementation

In-frame deletion of gene *relA* was generated by allelic exchange and named ∆*relA*. The *speA/relA* double deletion mutant was generated by deleting *speA* in the genetic background of the *relA* deletion mutant and designated as ∆*speA*∆*relA*. The detailed descriptions of strains and mutants are provided in Table S1. The flanking region of gene *relA* was amplified via PCR using the specific primers listed in Table S2. The PCR product was then digested with specific restriction enzymes, and cloned into suicide plasmid pKNG101 digested with the same enzymes. The resulting constructs were transformed into *E. coli* CC118 and then introduced into *D. oryzea* EC1 by triparental mating. For complementation analysis, the coding region of the target gene was amplified by PCR using specific primers by the primers listed in Table S2. The PCR products were digested with restriction enzymes and then cloned into the expression vector pBBR1-MCS4 digested with the same enzymes. The complementation constructs were introduced into corresponding mutants by tri-parental mating using the helper strain HB101 (pRK2013) and confirmed by PCR analysis.

### Motility assays

For swimming motility, 2 µL overnight cultures of *D. oryzea* and its derivatives were spotted inside the agar at the center of semisolid Bacto tryptone agar medium (each liter contains 10 g bacteriological peptone, 5 g NaCl, and 3 g agar). Plates were incubated at 28°C for 24 h and the diameters of swimming motility were measured. The swarming motility was assayed under the same conditions, except the semisolid medium containing (per liter) 5 g tryptone, 5 g NaCl, and 4 g agarose. To rescue the defective swimming motility of the mutants ∆*relA* and ∆*relA*∆*speA*, exogenous putrescine signal and alarmone ppGpp were added to corresponding media at a final concentration of 100 nM and 50 nM, respectively, and the swimming motility was quantified using the method described above. The experiments were repeated three times with triplicates.

### RNA extraction and real-time qPCR analysis

Overnight cultures of strain EC1 and derivatives were cultured in LS_5_ medium with putrescine at the final concentration of 100 nM or without at 28°C to OD_600_ = 1.0 or 1.5. The RNA samples were prepared using the SV total RNA isolated system kit (Promega) and further purified using the RNA clean kit (Qiagen). The qPCR analysis was performed using StarScript II first-strand cDNA synthesis Mix following the manufacturer’s protocol (GenStar) with the primers listed in Table S2. For RT-PCR analysis, the intergenic regions of *potF* and *plaP* were amplified for different samples using 2 ×  EasyTaq PCR SuperMix (+dye) (TransGen Biotech) following the manufacturer’s instructions. The qPCR analysis was conducted on a Quantstudio 6 Flex system using PowerUp SYBR green master mix (Thermo Fisher Scientific) with the following cycle profile: 1 cycle at 50°C for 2 min and 95°C for 2 min, followed by 45 cycles at 95°C for 15 s, 60°C for 15 s, and 72°C for 30 s. The experiment was repeated three times, each time with triplicates. Data were analyzed using the 2^−ΔΔCT^ method as previously described (55).

### Transcriptional fusion reporter system construction and flow cytometry analysis

The promoter region of genes *speA*, *potF*, *plaP,* and *relA* was predicted using the online tool provided by BPROM (http://www.softberry.com/berry.phtml?topicbprom&groupprograms&subgroup gfindb). The predicted promoter region of each gene was amplified using the specific primer pairs listed in Table S2 and ligated into the promoterless *gfp*-reporter plasmid pPROBE-NT (56). The fusion vector of pSpeA*_gfp_*, pPotF*_gfp_*, and pPlaP*_gfp_* was individually mobilized into wild-type EC1 and the *relA* deletion mutant by triparental mating with the helper strain HB101(pRK2013) to generate EC1(pSpeA*_gfp_*), EC1(pPotF*_gfp_*), EC1(pPlaP*_gfp_*), Δ*relA*(pSpeA*_gfp_*), Δ*relA*(pPotF*_gfp_*), and Δ*relA*(pPlaP*_gfp_*). Using a similar method, we also construed EC1(pRelA*_gfp_*), Δ*speA*(pRelA*_gfp_*), Δ*speA*(pPotF*_gfp_*), and Δ*speA*(pPlaP*_gfp_*). Expression of these genes was analyzed by monitoring the average fluorescence of 50,000 cells when bacteria were grown in flasks with swimming medium with or without exogenous addition putrescine signal and alarmone ppGpp at the final concentration of 100 nM and 50 nM by a CytoFLEX flow cytometer (Beckman Coulter, Brea, CA, USA) following the previously described method, respectively (57).

### Transposon mutagenesis and sequence analysis of *potF* expression deficient mutants

Mutagenesis experiments were performed using the mariner-based transposon system carried on plasmid pBT20 (58). Briefly, conjugal mating was performed by mixing overnight cultures of donor and recipient strains in about a 2:1 ratio onto LB agar plates and incubating at 28°C for 6 h. Tn5 mutants were selected on minimal medium agar plates containing Km, and the mutants were then screened for altered production of fluorescence using a microplate reader at 530 nm. Genomic DNA of selected mutants was extracted using the HiPure Bacterial DNA Kits (Magen, Guangzhou, China) and the flanking regions of transposon insertion sites were amplified by FPNI-PCR using the primers in Table S2 (59).

### Zeamine production assay

The assay of zeamine production was conducted by the spot-on-lawn assay as previously described. Briefly, 100 µL of *E. coli* DH5 fresh culture was added into 7.5 mL of 1% agarose, mixed and poured onto the surface of an LB agar plate (10 cm × 10 cm), dried at room temperature and punched with a 5 mm puncher and 30 mL of cell-free supernatants of *D. oryzea* EC1 (filter sterilized with a 0.22 m pore filter) were added in each well. The plates were incubated at 37°C for 24 h, and the inhibition zone around the wells was measured. For semi-quantification, one unit of zeamine was defined as the amount that could generate a 2-mm-diameter inhibitory zone around the well. Therefore, the number of zeamine units per milliliter was calculated by multiplying the units of zeamine calculated from the bioassay by the fold change of sample volume used in the assay (30 mL) to the total volume (1 mL).

### Rice seed germination assay

Determination of bacterial pathogenicity against rice seeds was performed following previously described methods (18) with minor modifications. Briefly, overnight bacterial cultures were diluted in 10-fold series, and the CFU of each dilution was determined using a heterotrophic plate counting assay. Thirty rice seeds were put into the tubes with 9 mL of bacterial dilution and incubated for 5 h in a room temperature. The rice seeds were rinsed three times with sterilized water and transferred onto two moistened filter papers in a petri dish. The seeds were then incubated at 28°C with a 16 h light and 8 h dark cycle, and sterilized water was added when necessary. The germination rate was determined 5 days after treatment. Rice seeds treated with sterilized water were used as a negative control. The experiment was repeated four times.

### Statistical analysis

All experiments were individually performed at least twice with three replicates each time. Statistical comparison was performed using the two-tailed unpaired Student’s *t* test in GraphPad Prism v.7.0 software (GraphPad). A *P* value of <0.05 was considered significant.

## SUPPLEMENTAL MATERIAL

Supplemental material msphere.00682-24-s0001.doc Tables S1 and S2; Figures S1 and S2.

ASM does not own the copyrights to Supplemental Material that may be linked to, or accessed through, an article. The authors have granted ASM a non-exclusive, world-wide license to publish the Supplemental Material files. Please contact the corresponding author directly for reuse.
