# Supplementary material for: Alarmone ppGpp modulates bacterial motility, zeamine production, and virulence of Dickeya oryzae through the regulation of and cooperation with the putrescine signaling mechanism
Source: mSphere. 2025 Mar 20;10(4):e00682-24. doi: 10.1128/msphere.00682-24 (PMC12039241; doi:10.1128/msphere.00682-24)
Supplement: Supplemental material — Tables S1 and S2; Figures S1 and S2. [file msphere.00682-24-s0001.doc]

**Supporting Information**

**Supplementary Table S1.** Strains and plasmids used in this study

| **Strains or plasmid** | **Relevant phenotypes and characteristics^a^** | **Source** |
| --- | --- | --- |
| ***Dickeya oryzea*** |  |  |
| EC1 | Wild type of *Dickeya oryzea*, Pmb ^r^ | Lab collection |
| ∆*speA* | *speA* deletion mutant derived from EC1 | Lab collection |
| ∆*relA*  ∆*speA*∆*relA*  ∆*relA*(*relA*)  ∆*speA*∆*relA*(*relA*)  ∆*speA*∆*relA*(*speA*)  ∆*speA*∆*relA*(*hfq*)  EC1(pSpeA*_gfp_*)  EC1(pRelA*_gfp_*)  EC1(pPotF*_gfp_*)  EC1(pPlaP*_gfp_*)  ∆*speA*(pRelA*_gfp_*)  ∆*speA*(pPotF*_gfp_*)  ∆*speA*(pPlaP*_gfp_*)  ∆*relA*(pSpeA*_gfp_*)  ∆*relA*(pPotF*_gfp_*)  ∆*relA*(pPlaP*_gfp_*) | *relA* deletion mutant derived from EC1  *speA* deletion mutant derived from ∆*relA*  The complemented strain of ∆*relA*, Amp^r^  The complemented strain of ∆*speA*∆*relA*, Amp^r^  The complemented strain of ∆*speA*∆*relA*, Amp^r^  Over-expression gene *hfq* strain of ∆*speA*∆*relA*, Amp^r^  EC1 containing pSpeA*_gfp_* vector, Kan^r^  EC1 containing pRelA*_gfp_* vector, Kan^r^  EC1 containing pPotF*_gfp_* vector, Kan^r^  EC1 containing pPlaP*_gfp_* vector, Kan^r^  ∆*speA* containing pRelA*_gfp_* vector, Kan^r^  ∆*speA* containing pPotF*_gfp_* vector, Kan^r^  ∆*speA* containing pPlaP*_gfp_* vector, Kan^r^  ∆*relA* containing pSpeA*_gfp_* vector, Kan^r^  ∆*relA* containing pPotF*_gfp_* vector, Kan^r^  ∆*relA* containing pPlaP*_gfp_* vector, Kan^r^ | This study  This study  This study  This study  This study  This study  This study  This study  This study  This study  This study  This study  This study  This study  This study  This study |
| ***Escherichia coli*** |  |  |
| CC118 | Host for plasmid constructs derived from pKNG101 | Lab collection |
| DH5α | Host for plasmid constructs derived from pBBRI-MCS4 | Lab collection |
| pRK2013 | *Thr leu thi recA hsdR hsdM pro,* Km^r^ | Lab collection |
| pBT20 | *E. coli* S17-λpir containing pBT20, Km^r^ | Lab collection |
| **Plasmids** |  |  |
| pKNG101 | Knockout vector, Str^r^ | Lab collection |
| pKNG101-*speA*  pKNG101-*relA* | pKNG101 containing in-frame deleted fragement of *speA*, Str^r^  pKNG101 containing in-frame deleted fragement of *relA*, Str^r^ | Lab collection  This study |
| pBBRI-MCS4 | Multi-copy expression vector, Gm^r^ | Lab collection |
| pBBRI- *speA*  pBBRI- *relA*  pBBRI- *hfq* | pBBRI-MCS4 containing *speA* encoding region at the downstream of *lac* promoter, Amp^r^  pBBRI-MCS4 containing *relA* encoding region at the downstream of *lac* promoter, Amp^r^  pBBRI-MCS4 containing *hfq* encoding region at the downstream of *lac* promoter, Amp^r^ | Lab collection  This study  Lab collection |
| pPROBE-NT | Promoterless *gfp* transcriptional reporter plasmid, Kan^r^ |  |
| pSpeA*_gfp_*  pRelA*_gfp_*  pPotF*_gfp_*  pPlaP*_gfp_* | *gfp* transcriptional fusion with upstream region of *speA* in EC1  *gfp* transcriptional fusion with upstream region of *relA* in EC1  *gfp* transcriptional fusion with upstream region of *potF* in EC1  *gfp* transcriptional fusion with upstream region of *plaP* in EC1 | This study  This study  This study  This study |

^a^ Pmb ^r^, Amp^r^, Km^r^, or Str^r^ = resistance to polymyxin B, ampicillin, kanamycin, or streptomycin, respectively.

**Supplementary Table S2.** Primers used in this study

| Primer | Decription^a^ | Sequence |
| --- | --- | --- |
| A-1 | Forward primer for upstream of *relA* | 5’-CGGGATCCCCGCTGCTGGATTTTATGCG-3’ |
| A-2 | Reverse primer for upstream of *relA* | 5’-GCGGTAAGAAGTGCGCATTTCCGGATGTGATTGACGCTCG-3’ |
| A-3 | Forward primer for downstream of *relA* | 5’-CGAGCGTCAATCACATCCGGAAATGCGCACTTCTTACCGC-3’ |
| A-4 | Reverse primer for downstream of *relA* | 5’-GGACTAGTGGCATGGGCAATTTCACGTT-3’ |
| HB-A-F | Forward primer for encoding region of *relA* | 5’-CGGAATTCGCGGGATAGCAAGGTGTTGA-3’ |
| HB-A-R | Reverse primer for encoding region of *relA* | 5’-CGGGATCCCGCCATACGTGCGTAAAACA-3’ |
| 16F | Forward primer for RT-PCR of 16S rRNA | 5’-GGCCTAACACATGCAAGTCG-3’ |
| 16R | Reverse primer for RT-PCR of 16S rRNA | 5’-GAGTTAGCCGGTGCTTCTTC-3’ |
| potF -F | Forward primer for RT-PCR of gene *potF* | 5’-CATAAGTACGCGGTGCCGTA-3’ |
| potF -R | Reverse primer for RT-PCR of gene *potF* | 5’-GGCTTCAACACCAGATCCCA-3’ |
| plaP-F | Forward primer for RT-PCR of gene *plaP* | 5’-TCATTCCTCGGCTTCGATGG-3’ |
| plaP-R | Reverse primer for RT-PCR of gene *plaP* | 5’-CCACAACGAAGATCACCCCA-3’ |
| zmsA-F | Forward primer for qPCR of gene *zmsA* | 5’-ATCGCAGATATCCGCAGTGG-3’ |
| zmsA-R | Reverse primer for qPCR of gene *zmsA* | 5’-CGTACCGTAGCCTGTGACTC-3’ |
| zmsB-F | Forward primer for qPCR of gene *zmsB* | 5’-CGCCGTTTAAGGCGATTGAG-3’ |
| zmsB-R | Reverse primer for qPCR of gene *zmsB* | 5’-GGTGATCCCACAGGACGTTT-3’ |
| zmsC-F | Forward primer for qPCR of gene *zmsC* | 5’-CGTCGGGTCAGTGATATCGG-3’ |
| zmsC-R | Reverse primer for qPCR of gene *zmsC* | 5’-CATCAGGTGTGCAGTGTTGC-3’ |
| zmsD-F | Forward primer for qPCR of gene *zmsD* | 5’-AGCAGGTGGATCCGCTTATG-3’ |
| zmsD-R | Reverse primer for qPCR of gene *zmsD* | 5’-GGGCTACCGCAGTAACACTT-3’ |
| zmsE-F | Forward primer for qPCR of gene *zmsE* | 5’-ACAGTGCTAGTGGGCGTTAC-3’ |
| zmsE-R | Reverse primer for qPCR of gene *zmsE* | 5’-AACGGAACGTCAACCCAGTT-3’ |
| rsmC-F | Forward primer for qPCR of gene *rsmC* | 5’-GTTCGGTTCGCTTACCAGGA-3’ |
| rsmC-R | Reverse primer for qPCR of gene *rsmC* | 5’-TGGAAAACGAAAGCGTGCTG-3’ |
| hfq-F | Forward primer for qPCR of gene *hfq* | 5’-ATACCGGGCGAGAAGGAACT-3’ |
| hfq-R | Reverse primer for qPCR of gene *hfq* | 5’-ACTGCAAGGCCAGATTGAGT-3’ |
| P-speA-F | Forward primer for pSpeA*_gfp_* construction | 5’-TGACAAGCTTCGCTTTTCTTCTGACGCCAC-3’ |
| P-speA-R | Reverse primer for pSpeA*_gfp_* construction | 5’-CACGGATCCGCGGTCATTCATGGCTACCT-3’ |
| P-relA-F | Forward primer for pRelA*_gfp_* construction | 5’-TGACAAGCTTGTGTGATGCCGCAGATTGTG-3’ |
| P-relA-R | Reverse primer for pRelA*_gfp_* construction | 5’-CACGGATCCGACGTTCGCACGACGTTTTA-3’ |
| P-potF-F | Forward primer for pPotF*_gfp_* construction | 5’-TGACAAGCTTGTTTGCCAGACATTCAGTGCC-3’ |
| P-potF-R | Reverse primer for pPotF*_gfp_* construction | 5’-CACGGATCCGACGCGGCCACCATTAAGC-3’ |
| P-plaP-F | Forward primer for pPlaP*_gfp_* construction | 5’-TGACAAGCTCTGAGCTTCAAATTCCCGACGTT-3’ |
| P-plaP-R | Reverse primer for pPlaP*_gfp_* construction | 5’-CACGGATCCTTGTACGCGTGATGTGGAAACC-3’ |
| FP1 | primer for sequence analysis of Tn5 mutants | 5’-GTAATACGACTCACTATAGGGCACGCGTGGTNTCGASTWTSGWGTT-3’ |
| FP2 | primer for sequence analysis of Tn5 mutants | 5’-GTAATACGACTCACTATAGGGCACGCGTGGTNGTCGASWGANAWGAA-3’ |
| FP3 | primer for sequence analysis of Tn5 mutants | 5’-GTAATACGACTCACTATAGGGCACGCGTGGTWGTGNAGWANCANAGA-3’ |
| FP4 | primer for sequence analysis of Tn5 mutants | 5’-GTAATACGACTCACTATAGGGCACGCGTGGTAGWGNAGWANCAWAGG-3’ |
| FP5 | primer for sequence analysis of Tn5 mutants | 5’-GTAATACGACTCACTATAGGGCACGCGTGGTNGTAWAASGTNTSCAA-3’ |
| FP6 | primer for sequence analysis of Tn5 mutants | 5’-GTAATACGACTCACTATAGGGCACGCGTGGTNGACGASWGANAWGAC-3’ |
| FP7 | primer for sequence analysis of Tn5 mutants | 5’-GTAATACGACTCACTATAGGGCACGCGTGGTNGACGASWGANAWGAA-3’ |
| FP8 | primer for sequence analysis of Tn5 mutants | 5’-GTAATACGACTCACTATAGGGCACGCGTGGTGTNCGASWCANAWGTT-3’ |
| FP9 | primer for sequence analysis of Tn5 mutants | 5’-GTAATACGACTCACTATAGGGCACGCGTGGTNCAGCTWSCTNTSCTT-3’ |
| FSP1 | primer for sequence analysis of Tn5 mutants | 5’-GTAATACGACTCACTATAGGGC-3’ |
| FSP2 | primer for sequence analysis of Tn5 mutants | 5’-ACTATAGGGCACGCGTGGT-3’ |
| SP1 | primer for sequence analysis of Tn5 mutants | 5’-TGTTACGCAGCAGGGCAGTCGC-3’ |
| SP2 | primer for sequence analysis of Tn5 mutants | 5’-CCTACTCCCAACATCAGCCGGACTC-3’ |
| SP3 | primer for sequence analysis of Tn5 mutants | 5’-TACGGTGACGATCCCGCAGT-3’ |

**
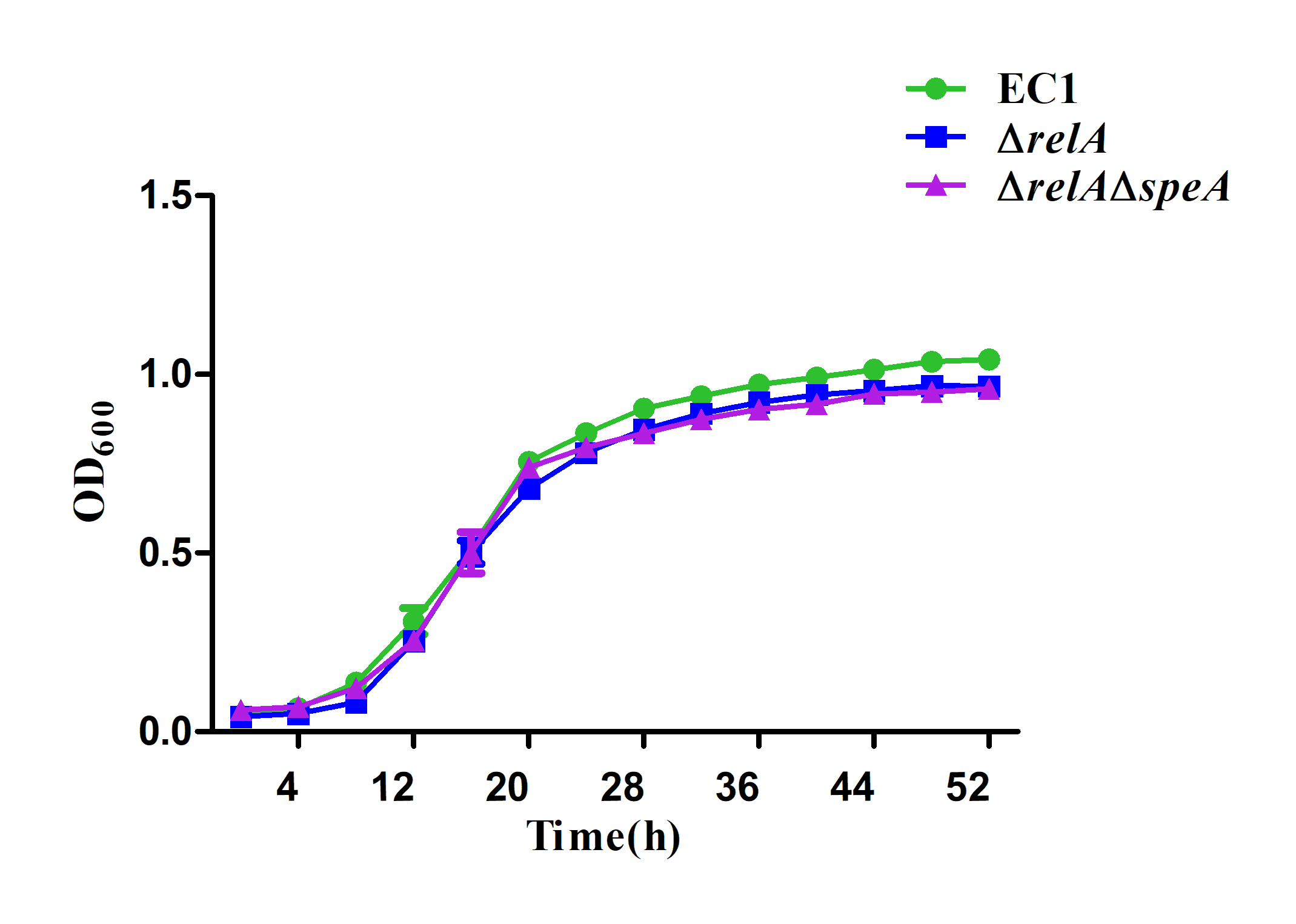
**

**Supplementary Figure S1.** Growth curves of *Dickeya oryzea* EC1 and its derivatives in the minimal medium. ∆*relA* indicated the *relA*-deletion mutant and ∆*relA*∆*speA* was the *speA*-deletion mutant in *relA*-deletion genetic background.

A

EC1

∆*relA*

∆*speA*


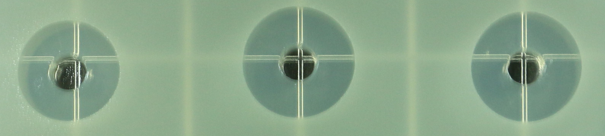


B

C

EC1

∆*relA*∆*speA*

∆*relA*∆*speA*

EC1


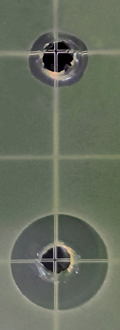

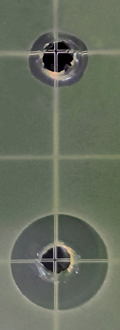


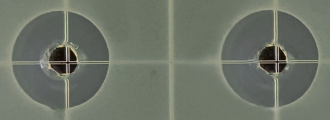

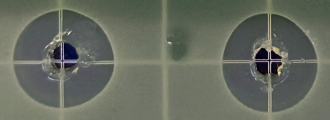


∆*relA*∆*speA*(*speA*)

∆*relA*∆*speA*(*relA*)

∆*relA*∆*speA*+Put

∆*relA*∆*speA*+ppGpp

D


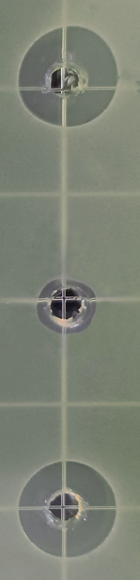


∆*relA*∆*speA*(*hfq*)

∆*relA*∆*speA*

EC1

**Supplementary Figure S2.** The plate assay of the antimicrobial activities of *Dickeya oryzea* EC1 and its derivatives. ∆*relA* and ∆*speA* indicated the deletion mutants of genes *relA* and *speA*, respectively. ∆*relA*∆*speA* indicated *relA-speA* double disrupted mutant, ∆*relA*∆*speA*(*relA*) and ∆*relA*∆*speA*(*speA*) indicated the complemented strains of mutant ∆*relA*∆*speA*, and ∆*relA*∆*speA+*Put and ∆*relA*∆*speA+*ppGpp indicated mutant ∆*relA*∆*speA* cultured with exogenous addition of 100 nM putrescine signal and 50 nM ppGpp signal, respectively. The photographs were taken after 16 h of incubation at 37 ℃.
